# *Glutamine synthetase* in Durum Wheat: Genotypic Variation and Relationship with Grain Protein Content

**DOI:** 10.3389/fpls.2016.00971

**Published:** 2016-07-13

**Authors:** Domenica Nigro, Stefania Fortunato, Stefania L. Giove, Annalisa Paradiso, Yong Q. Gu, Antonio Blanco, Maria C. de Pinto, Agata Gadaleta

**Affiliations:** ^1^Department of Soil, Plant and Food Sciences, University of Bari Aldo MoroBari, Italy; ^2^Department of Agricultural and Environmental Sciences, Research Unity of Genetic and Plant Biotechnology, University of Bari Aldo MoroBari, Italy; ^3^Department of Biology, University of Bari Aldo MoroBari, Italy; ^4^Crop Improvement and Genetics Research, Western Regional Research Center, United States Department of Agriculture – Agricultural Research Service, AlbanyCA, USA

**Keywords:** wheat, grain protein content, GS (Glutamine synthetase), qRT-PCR, enzyme activity, western blot

## Abstract

Grain protein content (GPC), is one of the most important trait in wheat and its characterized by a very complex genetic control. The identification of wheat varieties with high GPC (HGPC), as well as the characterization of central enzymes involved in these processes, are important for more sustainable agricultural practices. In this study, we focused on *Glutamine synthetase* (*GS*) as a candidate to study GPC in wheat. We analyzed *GS* expression and its enzymatic activity in different tissues and phenological stages in 10 durum wheat genotypes with different GPC. Although each genotype performed quite differently from the others, both because their genetic variability and their adaptability to specific environmental conditions, the highest GS activity and expression were found in genotypes with HGPC and *vice versa* the lowest ones in genotypes with low GPC (LGPC). Moreover, in genotypes contrasting in GPC bred at different nitrogen regimes (0, 60, 140 N Unit/ha) GS behaved differently in diverse organs. Nitrogen supplement increased *GS* expression and activity in roots of all genotypes, highlighting the key role of this enzyme in nitrogen assimilation and ammonium detoxification in roots. Otherwise, nitrogen treatments decreased GS expression and activity in the leaves of HGPC genotypes and did not affect GS in the leaves of LGPC genotypes. Finally, no changes in *GS* and soluble protein content occurred at the filling stage in the caryopses of all analyzed genotypes.

## Introduction

Global agriculture urgently requires a modification of standard breeding practices and management policies. A recent report by the United Nation (The Millennium Development Goals Report, 2014) highlighted that the world’s population reached 7.2 billion in 2014 and is expected to increase by more than 2 billion by 2050. This means that in the very near future even higher production will be needed to maintain food supplies. Indeed, breeders and scientists have focused their efforts on the identification of agricultural practices and the development of new genetic technologies.

Agricultural productivity had increased in recent decades through the diffusion of modern crop production practices, such as the spread of high-yielding crop varieties and a heavier use of mineral fertilizers. Nitrogen is the most important nutrient and, secondly only to water, a limiting factor for plant growth and development (Kraiser et al., 2011). In the last 40 years, the amount of nitrogen fertilizers supplied to crops has risen dramatically from 12 to 104 Tg/year (Mulvaney et al., 2009). This excess in synthetic N supply significantly affected yield increase. However, as reported in the statistics from the Food and Agricultural Organization of the United Nations, the yield of crops, especially wheat, soybean and maize, have slowed to a growth rate of about 1% annually, and in some specific cases, as in developed countries, the growth rate is quite close to zero (Fischer et al., 2009). Much of this nitrogen is wasted, as well – of the total amount of N supplied, only 30–50% is actually taken up by the plant (depending on the species and cultivar) and used in different biochemical pathways. Most is lost to the environment in several ways, such as surface run-off, leaching of nitrates, ammonia (NH_3_) volatilization or bacterial competition (Garnett et al., 2009). This represents a considerable expense both in terms of cost and environmental impact. Control directives and best management practices have been implemented several years ago to minimize environmental damage from nitrogen run-off (The Nitrates Directive, EC91/676/EEC, The EU Water Framework Directive, 2000/60/EC). Several studies and international projects have since highlighted the importance of defining the optimum timing and rate of nitrogen application during plant growth to maximize yield.

One of the most valuable agronomic and physiological indicators of how plants respond and use available N is nitrogen use efficiency (NUE), at first defined as the yield of grain per unit of available nitrogen in the soil (Moll et al., 1982, 1987). Currently, NUE could be defined as the ratio among plant grain yield and plant-available N in the soil, including soil-native N and N applied as fertilizer, and is composed of N-uptake efficiency and physiological N-use efficiency (De Macale and Velk, 2004). There is a need to diversify NUE significance, as there are several interpretations of this agronomic trait, depending on species and parameters of interest to be evaluated (Pathak et al., 2011; Hawkesford et al., 2013). Barraclough et al. (2014) studied how to quantify genetic variation in the uptake, portioning and remobilization of nitrogen in individual plant organs at extreme rates on N supply and can influence grain protein content (GPC). They found out that biggest contributor to variation in plant and crop performance was N-rate, followed by growth stage and finally genotype.

Glutamine synthetase (GS), an enzyme with an essential role in the assimilation of inorganic N, has been proposed as a candidate for improving NUE in wheat (Habash et al., 2007; Gadaleta et al., 2011, 2014; Thomsen et al., 2014). GS is present in most species, with three to five isoforms localized in the cytosol (GS1) and a single isoform (GS2) in plastids (Swarbreck et al., 2011). On the bases of phylogenetic studies and mapping data in wheat, 10 GS cDNA sequences were classified into four sub-families denominate *GS1* (a, b, and c), *GS2* (a, b, and c), *GSr* (1 and 2), and *GSe* (1 and 2; Bernard et al., 2008; Thomsen et al., 2014). Bernard et al. (2008) reported that QTLs for flag leaf and total GS activity were positively co-localized with QTLs for grain and stem nitrogen amount, but smaller correlations were established with loci for grain yield components; they identified QTLs for GS activity co-localized to a *GS2* gene mapped on chromosome 2A and to the *GSr* gene on 4A. Genetic studies in rice (Obara et al., 2004) and maize (Gallais and Hirel, 2004) demonstrated co-localizations of QTLs for GS protein or activity with QTLs relating to grain parameters at the mapped GS genes.

To date few studies are available on the role of genotypic variation of GS for GPC. In this work, we present data on total GS activity and expression in 10 wheat genotypes in relation to their final GPC. Moreover, the response to nitrogen supplies in terms of total *GS* expression and activity of four different wheat genotypes, differing in GPC, has been investigated.

## Materials and Methods

### Plant Material and Field Experiment Design

Ten different durum wheat genotypes (the breeding lines PI191145 and PC32, and genotypes Svevo, Cannizzo, Gianni, Ciccio, Appio, Lucanica, Canyon, and Vesuvio) were chosen from a collection of tetraploid wheat genotypes described by Laidò et al. (2013) and Marcotuli et al. (2015). Wheat genotypes were grown for 6 years (2009–2013) without any external nitrogen supply at Valenzano (Bari, Italy); geographical coordinates: 41° 2′ 0″ North, 16° 53′ 0″ East. A randomized complete block design with three replications and plots consisting in 2.0 m × 1.5 m, with a seed density of 350 germinated seeds/m^2^. According to the standard agronomic practices in the study’s area, fertilizer applications were made at pre-sowing (90 kg/ha P_2_O_5_). During the growing season standard cultivation practices were adopted without water supply. The plants were harvested after physiological maturity, on July 10, of each year.

The different selected genotypes were chosen according to previous evaluation of yield and quality component trait (unpublished data). In order to evaluated the involvement of candidate enzymes and genes only in the accumulation of GPC, genotypes with similar value of grain yield per spike (GYS) and thousand kernel weight (TKW) were chosen, in order to avoid the negative correlation between GPC and GYS or dilution factor due to TKW (Supplemetary Table S1).

In 2014, four genotypes (PC32, Cannizzo, Ciccio, and Vesuvio) were grown in Valenzano (BA) at three different nitrogen regimes: 0, 60, and 140 N Unit/ha in randomized blocks with replicates (indicated as N0, N60, and N140). Each genotype was sown in one linear meter row and 20 cm apart. Nitrogen was supplied, in the form of ammonia nitrate, in three equal rates, 10 days before collecting samples at stages of first leaf, flowering, and grain filling. Roots were collected from plants at the seedling stage immediately washed, removed excess of water, frozen in liquid nitrogen, and stored at -80°C. Leaf tissues of each sample were collected in each phase 10 days after nitrogen implementation, immediately frozen in liquid nitrogen, and stored at -80°C until used in further assays.

### Protein Content Quantification

Total GPC was assessed on 3 g of whole meal flour using a dual beam near infrared reflectance spectrophotometer (Zeutec Spectra Alyzer Premium, Zeutec Büchi, Rendsburg, Germany). Soluble proteins were assayed according to Bradford (1976), using bovine albumin as a standard.

### GS Activity Determination

Plant tissues were frozen in liquid N and ground in a mortar with 1:10 (w/v) extraction buffer (100 mM triethanolamine, 1 mM EDTA, 10 mM MgSO_4_, 5 mM glutamate, 10% v/v ethylene glycol, 10 μM leupeptin and 6 mM DTT- pH7.6). Crude extracts were centrifuged at 21000 × *g* for 30 min at 4°C and the supernatant used for GS activity determination. GS activity was measured according to Bernard et al. (2008).

### GS Immunoblotting

The soluble proteins in each extract were separated through SDS-PAGE (Laemmli, 1970). Equal concentrations of denatured proteins (5 μg) were loaded in each track of a 12% polyacrylamide gel. The proteins were electrophoretically transferred to an ImmunoBlot PVDF membrane (Bio-Rad, München, Germany) with a *Trans*-Blot Semi-Dry (Bio-Rad, München, Germany) by using a transfer buffer containing 25 mM Tris, 190 mM Glycine, 20% methanol. The electrophoretic transfer was conducted at 15 V for 60 min. After the transfer, the PVDF membrane was soaked in blocking solution (20 mM Tris-HCl pH 7.5, 150 mM NaCl, 0,05% Tween 20, and 1% BSA) for 30 min; incubated overnight with primary antibody, and incubated with secondary antibody for 60 min. GS proteins were detected with GS1/GS2 glutamine synthetase global primary polyclonal antibody (Agrisera Vännäs, Sweden); which recognizes both cytoplasmic and chloroplastic forms of the GS enzyme. The secondary antibody was Anti-Rabbit IgG (H+L) HRP conjugate (Promega, Madison, WI, USA). The antibody-protein complex was detected with enhanced chemiluminescence (ECL)-detection reagents (Amersham Buchler Ltd); ImmunoBlot PVDF membrane was incubated for 2 min in ECL, then exposed in an X-ray cassette with Amersham Hyperfilm ECL (Amersham Buchler Ltd) for 2 min. The hyperfilm was soaked in a developing and fixing solution (Kodak Inc.).

### Quantitative RT-PCR

Total RNA was extracted with the RNeasy Plant Mini Kit (QIAGEN^®^), and checked on 1.5% denaturing agarose gels. The total amount of RNA and its purity was determined using a Nano-Drop ND1000 spectrophotometer (Thermo Scientific, Walthman, MA, USA). All RNA samples were adjusted to the same concentration (1 μg) for subsequent treatment with recombinant DNase I (Roche Applied Science, Mannheim, Germany) to remove genomic DNA, and then reverse-transcribed into double stranded cDNA with the Transcriptor First Strand cDNA Synthesis Kit (Roche Applied Science, Mannheim, Germany).

Data were normalized using three reference genes: Cell Division Control AAA-Superfamily of ATPases (CDC), ADP-Ribosylation Factor (ADP-RF), and RNase L Inhibitor-like protein (RLI; Paolacci et al., 2009; Giménez et al., 2011). These genes were previously used as references in other wheat gene expression studies (Nigro et al., 2013); all three have a stability value around 0.035 when evaluated with NormFinder software (Andersen et al., 2004). In order to pick a primer combination which could detect total GS expression, sequences of known GS genes were aligned in order to find conserved regions. Specifically, cDNAs sequences of both plastidic *GS2* (DQ124212, DQ124213 and DQ124214) and cytosolic isoforms *GS1* (DQ124209, DQ124210 and DQ124211), *GSe* (AY491970 and AY491971), and *GSr* (AY491968 and AY491969), reported by Bernard et al. (2008), were aligned and compared (**Supplementary Figure S1**). The primer combination was chosen in the region with higher homology among them, in particular a fragment of 149 bp (F 5′–3′: CCCTGGCCCCCAGGGTCCATACTACTG; R 5′–3′: GTCATGCCTGGTCAGTGGGAGT).

Quantitative Real-Time PCR analyses to determine *GS* genes expression levels were carried out using EVA GREEN^®^ in the CFX96^TM^ Real-Time PCR System (Bio-rad). The PCR cycle was 95°C for 3 min, followed by 40 cycles of 95°C for 10 s, 60°C for 30 s. Amplification efficiency (98–100%) for the primer set was determined by amplification of cDNA with a series of six scalar dilutions (1:5) per reaction. Each 10 μl PCR reaction contained 1 μl of a 1:5 dilution of cDNA, 5 μl of EvaGreen Mix 10X (Bio-Rad), and 500 nM of each primer. All experiments were performed in Hard-Shell 96-well skirted PCR plates (HSP9601) with Microseal^®^ ‘B’ Adhesive Seals (MSB-1001) from Bio-Rad. Fluorescence signals were recorded each cycle. The specificity of each amplicon was confirmed by the presence of a single band of the expected size during agarose gel electrophoresis (2% w/v), single peak melting curves of the PCR products, and sequencing of the amplified fragment. qRT-PCR data for both GS and endogenous controls genes are derived from the mean values of three independent amplification reactions carried out on five different plants harvested in the same phenotypic stage (biological replicates). All calculations and analyses were performed using CFX Manager 2.1 software (Bio-Rad Laboratories) using the Δ*C*t method, which uses the relative quantity (RQ) calculated with a ratio of the RQ of the target gene to the relative expression of the reference gene (including the three reference targets in each sample). Standard deviations were used to normalize values for the highest or lowest individual expression levels (CFX Manager 2.1 software user manual, Bio-Rad Laboratories).

### Statistical Analysis

Values are expressed as mean ± SEM. The medium values reported for GPC, GS activity, and GS expression in the high (H) and low (L) GPC groups were obtained mediating the values of all genotypes belonging to each group (Lucanica, PI191145, PC32, Cannizzo and Svevo for the HGPC; Ciccio, Vesuvio, Appio, Gianni, and Canyon for LGPC). One-way analysis of variance was conducted to calculate differences within and among the groups and for each treatment.

The Dumm’s test was used for comparisons among 10 genotypes with no treatment.

The Tukey’s test was used for comparisons among four treated genotypes. Correlations were calculated using the Spearman test. Differences were considered significant at *P*-values <0.05 (two-tailed). Analyses were performed using Sigma Plot software 12.0 (Systat Software, Inc., San Jose, CA, USA).

## Results

### Glutamine Synthetase Activity and Expression in 10 Durum Wheat Genotypes

**FIGURE 1:**
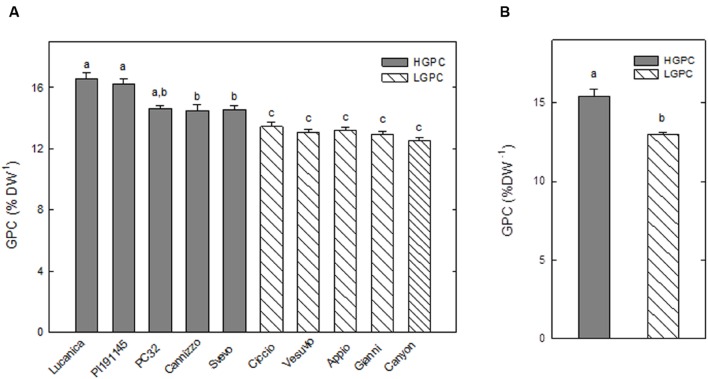
**Grain protein content (GPC) of 10 *Triticum durum* genotypes.** **(A)** GPC in 10 wheat genotypes cultivated for 5 years (2009–2013). Values are the mean ± SE of the medium value obtained in each year; different letters indicate significant differences (one-way ANOVA test; *P* < 0.05). **(B)** Average values of GPC for the HGPC and LGPC subgroups; Data are the means ± SE of the GPC of the cultivars belonging to HGPC and LGPC subgroups; different letters indicate values significantly different from one another (one-way ANOVA; *P* < 0.05).

The GPC, expressed as percentage of protein per dry weight, was analyzed for five consecutive years from 10 wheat genotypes. The genotypes were classified into either high GPC (HGPC: Lucanica, PI191145, PC32, Cannizzo, and Svevo), or low GPC (LGPC: Ciccio, Vesuvio, Appio, Gianni, and Canyon) groups. The average GPC values of the two groups were significantly different (**Figure 1**).

Both enzyme activity and gene expression of GS, a candidate gene for NUE and GPC, were analyzed in roots and leaves at different phenological stages in the 10 selected wheat genotypes grown in the field during the 2014 season.

**FIGURE 2:**
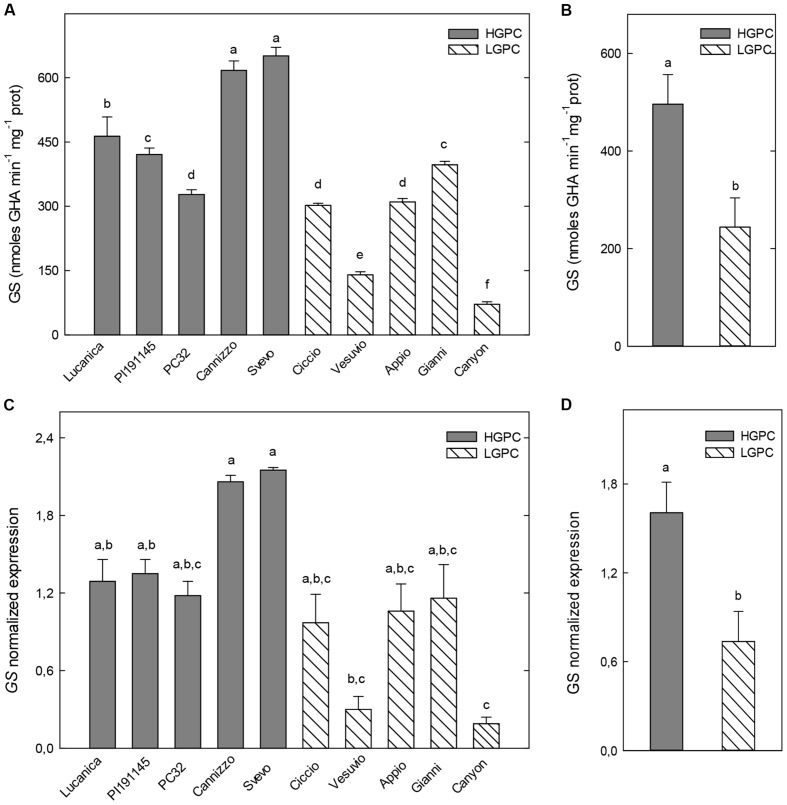
**Glutamine synthetase (GS) activity and expression in roots of 10 durum wheat genotypes differing in GPC.** **(A)** GS activity in 10 wheat genotypes. The mean (±SE; *n* = 5) is presented with different letters representing significant differences (one-way ANOVA test; *P* < 0.05). **(B)** GS activity in HGPC and LGPC subgroups; Data are the means ± SE of the GS activity of the cultivars belonging to HGPC and LGPC subgroups; different letters indicate significant differences (one-way ANOVA test; *P* < 0.05). **(C)** *GS* normalized fold expression in 10 wheat genotypes The mean (±SE; *n* = 5) is presented with different letters representing significant differences (*P* < 0.05). **(D)** *GS* expression in HGPC and LGPC subgroups; Data are the means ± SE of the GS activity of the cultivars belonging to HGPC and LGPC subgroups; different letters indicate significant differences (one-way ANOVA test; *P* < 0.05).

Glutamine synthetase activity in roots significantly differ among genotypes. However, the highest GS activities were found in two HGPC genotypes (Cannizzo and Svevo) and the lowest ones in the LGPC genotypes Vesuvio and Canyon (**Figure 2A**). As a consequence the overall mean of GS specific activity of HGPC genotypes was significantly higher than the average value of LGPC genotypes (**Figure 2B**). Expression data of GS in the roots of each cultivar were consistent with enzyme activity (**Figure 2C**) and again an higher mean value of GS expression was found in the HGPC group when compared with the LGPC one (**Figure 2D**).

**FIGURE 3:**
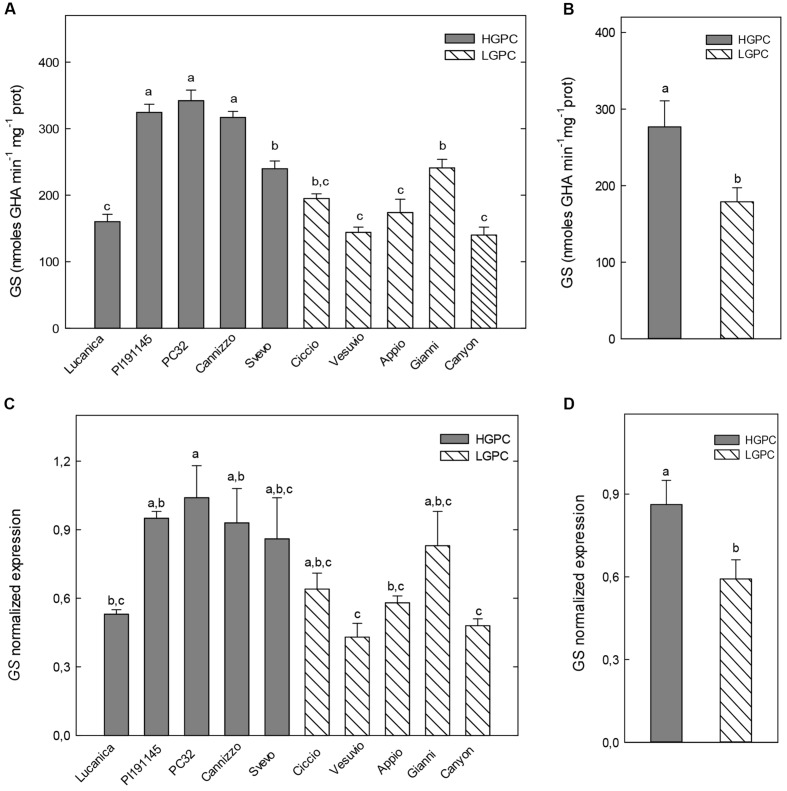
**Glutamine synthetase (GS) activity and expression in leaves at the first leaf stage of 10 durum wheat genotypes differing in GPC.** **(A)** GS activity in 10 wheat genotypes. The mean (±SE; *n* = 5) is presented with different letters representing significant differences (one-way ANOVA test; *P* < 0.05). **(B)** GS activity in HGPC and LGPC subgroups; Data are the means ± SE of the GS activity of the cultivars belonging to HGPC and LGPC subgroups; different letters indicate significant differences (one-way ANOVA test; *P* < 0.05). **(C)** *GS* normalized fold expression in 10 wheat genotypes. The mean (±SE; *n* = 5) is presented with different letters representing significant differences (*P* < 0.05). **(D)** *GS* expression in HGPC and LGPC subgroups; Data are the means ± SE of the GS activity of the cultivars belonging to HGPC and LGPC subgroups; different letters indicate significant differences (one-way ANOVA test; *P* < 0.05).

Similar trends were observed for GS activity and expression in the leaves at the first leaf stage. Indeed, although differences were found among GS activity and expression of each cultivar, the highest values were found in the HGPC group (PI191145, PC32, and Cannizzo) and the lowest ones in the LGPC group (Vesuvio, Appio, and Canyon; **Figures 3A,C**). Also in this case the medium values of enzyme activity and expression for HGPC genotypes, were significantly higher than that observed in LGPC genotypes (**Figures 3B,D**).

**FIGURE 4:**
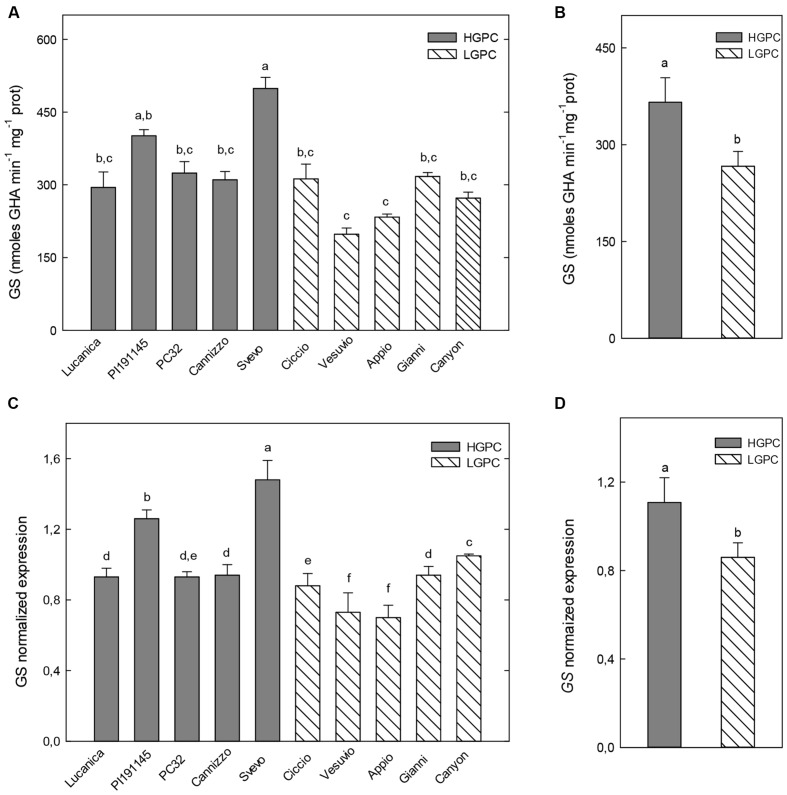
**Glutamine synthetase activity and expression in leaves at the flowering stage of 10 durum wheat genotypes differing in GPC.** **(A)** GS activity in 10 wheat genotypes. The mean (±SE; *n* = 5) is presented with different letters representing significant differences (one-way ANOVA test; *P* < 0.05). **(B)** GS activity in HGPC and LGPC subgroups; Data are the means ± SE of the GS activity of the cultivars belonging to HGPC and LGPC subgroups; different letters indicate significant differences (one-way ANOVA test; *P* < 0.05). **(C)** *GS* normalized fold expression in 10 wheat genotypes. The mean (±SE; *n* = 5) is presented with different letters representing significant differences (*P* < 0.05). **(D)** *GS* expression in HGPC and LGPC subgroups; Data are the means ± SE of the GS activity of the cultivars belonging to HGPC and LGPC subgroups; different letters indicate significant differences (one-way ANOVA test; *P* < 0.05).

At the flowering stage, differences in GS activity and expression in the leaves of the different genotypes were less marked, even if, also in this case, two HGPC genotypes (PI191145 and Svevo) showed the highest values and two LGPC genotypes (Vesuvio and Appio) the lowest ones (**Figures 4A,C**); the overall mean of GS activity and expression of HGPC genotypes resulted higher than that observed in LGPC genotypes (**Figures 4B,D**).

**FIGURE 5:**
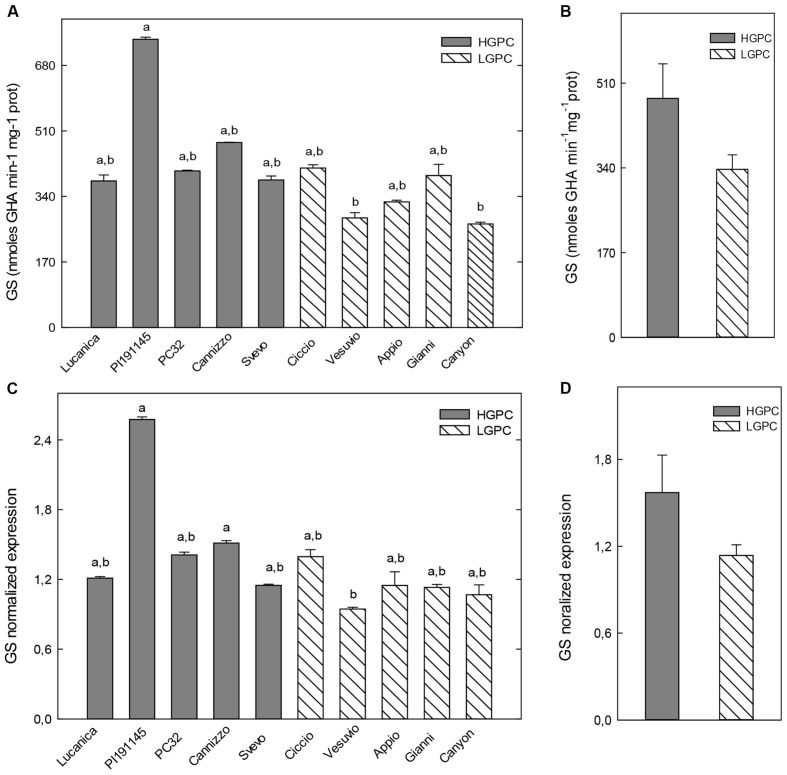
**Glutamine synthetase activity and expression in caryopses at the filling stage of 10 durum wheat genotypes differing in GPC.** **(A)** GS activity in 10 wheat genotypes. The mean (±SE; *n* = 5) is presented with different letters representing significant differences (one-way ANOVA test; *P* < 0.05). **(B)** GS activity in HGPC and LGPC subgroups; Data are the means ± SE of the GS activity of the cultivars belonging to HGPC and LGPC subgroups; different letters indicate significant differences (one-way ANOVA test; *P* < 0.05). **(C)** *GS* normalized fold expression in 10 wheat genotypes The mean (±SE; *n* = 5) is presented with different letters representing significant differences (*P* < 0.05). **(D)** *GS* expression in HGPC and LGPC subgroups. Data are the means ± SE of the GS activity of the cultivars belonging to HGPC and LGPC subgroups; different letters indicate significant differences (one-way ANOVA test; *P* < 0.05).

In the caryopses at the filling stage, the activity and the expression of GS did not change significantly among the genotypes of the two groups, with the exception of PI191145 that showed the highest values, and Vesuvio that had the lowest ones (**Figures 5A,C**). In this case the average values of *GS* activity and expression in the HGPC and LGPC groups did not differ significantly (**Figures 5B,D**).

Regression analysis conducted between GPC, enzymatic activity, and gene expression revealed significant correlation and were reported in **Table 1**.

**Table 1:** Coefficients of correlation (*R*^2^) and probability (*P*-value) between GPC, enzymatic activity, and gene expression.

Trait	*R*^2^	*P*
GPC-REA	0.35	0.05
GPC-CEA	0.42	0.05
GPC-CGE	0.36	0.05
REA-RGE	0.97	0.001
LIEA-LIGE	0.94	0.001
LIIEA-LIIGE	0.87	0.001
CEA-CGE	0.94	0.001


*GPC, grain protein content; REA, root enzymatic activity; RGE, root gene expression; LIEA, leaf I stage enzymatic activity; LIGE, leaf I stage gene expression; LIIEA, leaf II stage enzymatic activity; LIIGE, leaf II stage gene expression; CEA, caryopsis enzymatic activity; CGE, caryopsis gene expression. Significance at *P* ≤ 0.05, *P* ≤ 0.01, and *P* ≤ 0.001 levels, respectively.*

### Effect of Nitrogen Treatments on GS Activity and Expression in Different Wheat Genotypes

Two wheat genotypes from each group (PC32 and Cannizzo from the HGPC and Ciccio and Vesuvio from the LGPC) were grown in 2014 at Valenzano (BA) under three different rates of nitrogen application, N0, N60, and N140 units/ha (see Mat and Meth). GS activity and expression were followed in roots, leaves and caryopses of the four selected genotypes grown at different N fertilization.

Root GS activity increased in all genotypes after the N treatment (**Supplementary Figure S2**). However, in the HGPC genotypes PC32 and Cannizzo, the maximum increase in GS was already evident after application of 60 N units/ha and no further increase occurred after application of 140 N units/ha. The LGPC genotypes, Ciccio and Vesuvio, behaved differently. Ciccio increased root GS activity proportionally to the N application, whereas only the application of 140 N units/ha increased GS activity in Vesuvio roots (**Supplementary Figure S2A**). GS expression in roots had almost the same behavior of GS activity: a maximum increase in gene expression was observed after the application of 60 N units/ha in PC32 and Cannizzo; Ciccio showed an increase of root GS expression proportional to nitrogen supply and Vesuvio had no significant differences between gene expression at N0 and N60, but a significant increase occurred when 140 N units/ha was supplied (**Supplementary Figure S2B**).

The western blot analysis show the presence of a 40 kDa band in the four genotypes, indicating that only the cytosolic GS isoenzyme was present in the roots. The band intensity in the three different N treatments was consistent with GS transcript level and activity (**Supplementary Figure S2C**).

In leaf tissues at the first leaf stage, GS activity and expression significantly decreased with nitrogen application (N60 and N140 units/ha) in the two HGPC genotypes (PC32 and Cannizzo). On the other hand, nitrogen did not significantly change GS activity and expression in the Ciccio and Vesuvio genotypes (**Supplementary Figures S3A,B**). The Western blot analysis highlighted the presence of two bands of 44 and 40 kDa, indicating that both plastidic and cytosolic isoenzymes, respectively, were active in the leaves. Moreover, GS activity in the leaves at this stage seemed to be principally due to the plastidic GS, that was more abundant compared to the cytosolic one. Consistently with GS activity and expression, the intensity of the bands of GS proteins after the application of 140 N units/ha decreased in PC32 and Cannizzo and did not show differences in Ciccio and Vesuvio (**Supplementary Figure S3C**).

Glutamine synthetase activity in the leaves at the flowering stage was similar to that observed in the first leaf stage. In PC32 and Cannizzo, GS activity was still highest when no nitrogen was supplied, and decreased significantly with nitrogen applications. On the other hand, Ciccio and Vesuvio genotypes did not show significant differences in GS activity in all N regimes (**Supplementary Figure S4A**). RT-PCR analysis and western blot showed a decrease in the transcript and protein levels only in the PC32 genotype after application of 140 N units/ha (**Supplementary Figures S4B,C**).

Glutamine synthetase activity in the caryopses at the filling stage significantly increased only in the Vesuvio cultivar, which has the lowest GPC (**Supplementary Figure S5A**). However, when soluble GPC was measured in the three analyzed genotypes under different N treatment, no statistically significant differences were observed in the four genotypes after N supplies (**Supplementary Figure S5B**), indicating that final GPC was not affected by nitrogen application.

## Discussion

Nitrogen uptake and utilization is a very complex process in plants, and deciphering all its components is a challenge for scientists and breeders (Hawkesford et al., 2013). The quantitative traits of NUE and GPC are influenced both by the actions of multiple genes and environmental influence (Blanco et al., 2012). In the present work, the enzyme activity and expression of *GS*, a candidate gene for N-utilization efficiency, were studied in wheat in order to define its role in NUE and GPC. Genetic studies on NUE in maize and rice have shown that GS activity of a cytosolic GS isoform 1 co-localized with QTLs for N remobilization and grain size (Gallais and Hirel, 2004; Obara et al., 2004). In addition, rice mutants lacking the cytosolic GS gene OsGS1;1 were severely limited in growth and grain filling (Tabuchi et al., 2005). In *Triticum aestivum* a QTL for leaf GS activity, mapped to the *TaGSr* locus, co-localized with a QTL for grain N concentration. In this case, increased GS activity was associated with higher grain N. Phenotypic and genotypic correlations between flag leaf weight, soluble protein content and GS activity suggest shared control of leaf size and metabolic capacity during grain filling in wheat (Habash et al., 2007). Other two wheat *GS* genes, the plastidic *GS2* and the cytosolic *GS1.3*, have been associated with QTLs for GPC (Gadaleta et al., 2011, 2014). Moreover, in winter wheat GPC is positively correlated with amino acid and soluble protein content, and with GS activity (Fontaine et al., 2009). Our results show that a clear genotypic variation in GS activity and expression occurs in roots and leaves of the 10 durum wheat genotypes analyzed. However, despite the genotypic variation, the highest GS activities and expression have been found in genotypes of the HGPC group and *vice versa* the lowest ones in the genotypes of the LGPC group. As a consequence, GS activity and expression are on average higher in the HGPC group than in the LGPC one. Another study on five wheat cultivars exhibiting different NUE showed a good correlation between GS activity and the amount of N re-mobilized from the top section of the plant, or even from the flag leaf alone, to the grain (Kichey et al., 2006).

The situation is different in the caryopses at the filling stage, where no significant differences in GS activity and expression between the LGPC and HGPC genotypes were observed. This suggests that GS could be related to the maintenance of critical N flows and sensing during crucial developmental stages, as proposed by Thomsen et al. (2014).

To assess the effect of GS on NUE and GPC, four wheat genotypes were grown under different nitrogen regimes in field conditions. The obtained results are reasonably different for roots and leaves. In roots of all selected wheat genotypes, only cytosolic GS was present. Moreover, after N supply, an increase in GS expression and activity occurred both in the HGPC and LGPC genotypes. These data are consistent with results obtained in *Arabidopsis*; in roots, cytosolic GS is essential for ammonium detoxification and nitrogen assimilation under ample nitrate supply (Lothier et al., 2011). In rice, most of the ammonium taken up by the roots can be assimilated within the organ, as shown by the rapid up-regulation of *OsGS1;2* in the cell layers of the root surface following the supply of ammonium ions (Tabuchi et al., 2007).

The results are quite different in leaves at both the phenological stages considered. In accordance with what reported by Bernard et al. (2008), both plastidic and cytosolic enzymes were detected by Western blot, as two proteins of 44 and 40 kDa, respectively. After supplying nitrogen, total GS activity and expression in leaves did not change in the LGPC genotypes (Ciccio and Vesuvio) and significantly decreased in the HGPC genotypes (Cannizzo and PC32). These results are in accordance with data reported by Tian et al. (2015) showing that the expression of GS genes was higher in the N-efficient wheat genotype than in the N-inefficient one regardless of N treatment. Soluble GPC was not statistically significant in our genotypes after N treatments, implying that the genetic difference between cultivars caused differences in GPC. This is also consistent with the results by Gaju et al. (2011), who in analyzing fourteen UK and French wheat cultivars and two French advanced breeding lines showed that genetic variability in NUE related mainly to differences in N-utilization efficiency, rather than N-uptake efficiency.

Previous studies have reported that when NUE is calculated as a function of grain yield per estimated N input, this decreases with the increasing N input (Grant et al., 1991; Muurinen et al., 2007; Sylvester-Bradley and Kindred, 2009; Anbessa and Juskiw, 2012). The total N uptake of each cultivar in these studies was quite similar, implying that the differences observed in terms of grain yield in response to different N regimes and NUE was best assessed as differences in the efficiency of utilization. This suggests that the rate of nitrogen fertilizer application might be adjusted according to the individual cultivar to improve NUE, while maintaining potential grain yield.

Nitrogen use efficiency is a complex trait that cannot be explained by the action of a single gene. In a recent study on 24 Australian spring wheat genotypes, Mahjourimajd et al. (2016) analyzed how nitrogen supplies can affect NUE and yield in different environmental conditions. They demonstrated that there was significant genetic variation for NUE-related traits among wheat genotypes, allowing them to define a ranking of genotypes for NUE stability. Focusing and explaining the genetic mechanisms underlying traits associated with NUE are essential to contribute to wheat breeding efforts in order to develop high NUE genotypes. In this context, our data contribute to highlight that NUE is a genotype-dependent parameter, and that GS plays a very important role in terms of N utilization. So far, these studies confirm that the efficient management of N through the use of appropriate germplasm is essential for sustainability of agricultural production and that the use of genotypes optimized for traits relating to N-use efficiency rather than yield alone is of primary importance (Hawkesford, 2014). In this view, a more “precision farming” approach could be helpful to guarantee high grain yield while wasting little fertilizer, leading to both economic and environmental benefits.

## Author Contributions

DN, AG, and MP: Conceived and designed the experiments. DN, SF, SG, and AP: Performed the experiments. AG, AB, and MP: Contributed reagents/materials/analysis tools. DN, AG, MP, YG, and AB: Wrote the paper. DN, AG, MP, YG, and AB: Analyzed the data.

## Conflict of Interest Statement

The authors declare that the research was conducted in the absence of any commercial or financial relationships that could be construed as a potential conflict of interest.

## Supplementary Material

**FIGURE S1 | Alignment of glutamine synthetase (GS) genes region chosen for RT-PCR primer design.**

**FIGURE S2 | Glutamine synthetase (GS) in roots of four selected wheat genotypes bred at three different nitrogen rates.** **(A)** GS specific activity; **(B)** GS normalized fold expression; **(A)** GS specific activity and **(B)** GS expression; Data are the means ± SE of five experiments; different letters indicate significant differences after N treatment in each cultivar (one-way ANOVA test; *P* < 0.05). **(C)** Representative image of western blotting analysis with the GS1/GS2 antibody; each well was loaded with 5 μg soluble proteins.

**FIGURE S3 | Glutamine synthetase in leaves at the first leaf stage, of four selected wheat genotypes bred at three different nitrogen rates.** **(A)** GS specific activity and **(B)** GS expression; data are the means ± SE of five experiments; different letters indicate significant differences after N treatment in each cultivar (one-way ANOVA test; *P* < 0.05). **(C)** Representative image of western blotting analysis with the GS1/GS2 antibody; each well was loaded with 5 μg soluble proteins.

**FIGURE S4 | Glutamine synthetase in leaves at the flowering leaf stage, of four selected wheat genotypes bred at three different nitrogen rates.** **(A)** GS specific activity and **(B)** GS expression; data are the means ± SE of five experiments; different letters indicate significant differences after N treatment in each cultivar (one-way ANOVA test; *P* < 0.05). **(C)** Representative image of western blotting analysis with the GS1/GS2 antibody; each well was loaded with 5 μg soluble proteins.

**FIGURE S5 | Glutamine synthetase and soluble protein content in caryopses at the filling stage of four selected wheat genotypes bred at three different nitrogen rates.** **(A)** GS activity and **(B)** soluble protein content. Data are the means ± SE of five experiments; different letters indicate significant differences after N treatment in each cultivar (one-way ANOVA test; *P* < 0.05).
