# Supplementary figures and images for: Glutamine synthetase in Durum Wheat: Genotypic Variation and Relationship with Grain Protein Content
Source: Front Plant Sci. 2016 Jul 13;7:971. doi: 10.3389/fpls.2016.00971 (PMC4942471; doi:10.3389/fpls.2016.00971)

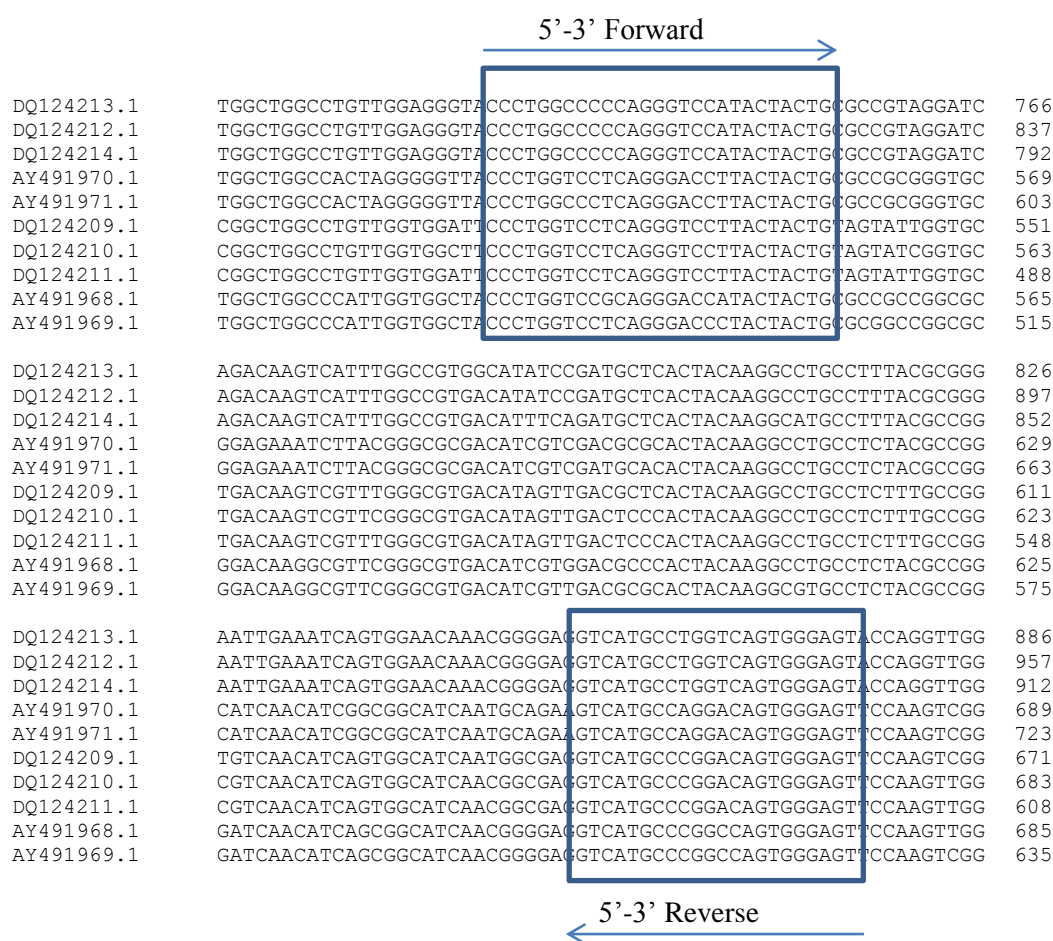

**Supplementary Figure 1.** Alignment of GS genes region chosen for RT-PCR primer design

Supplement: Supplementary file 1 [file Image_1.PDF]

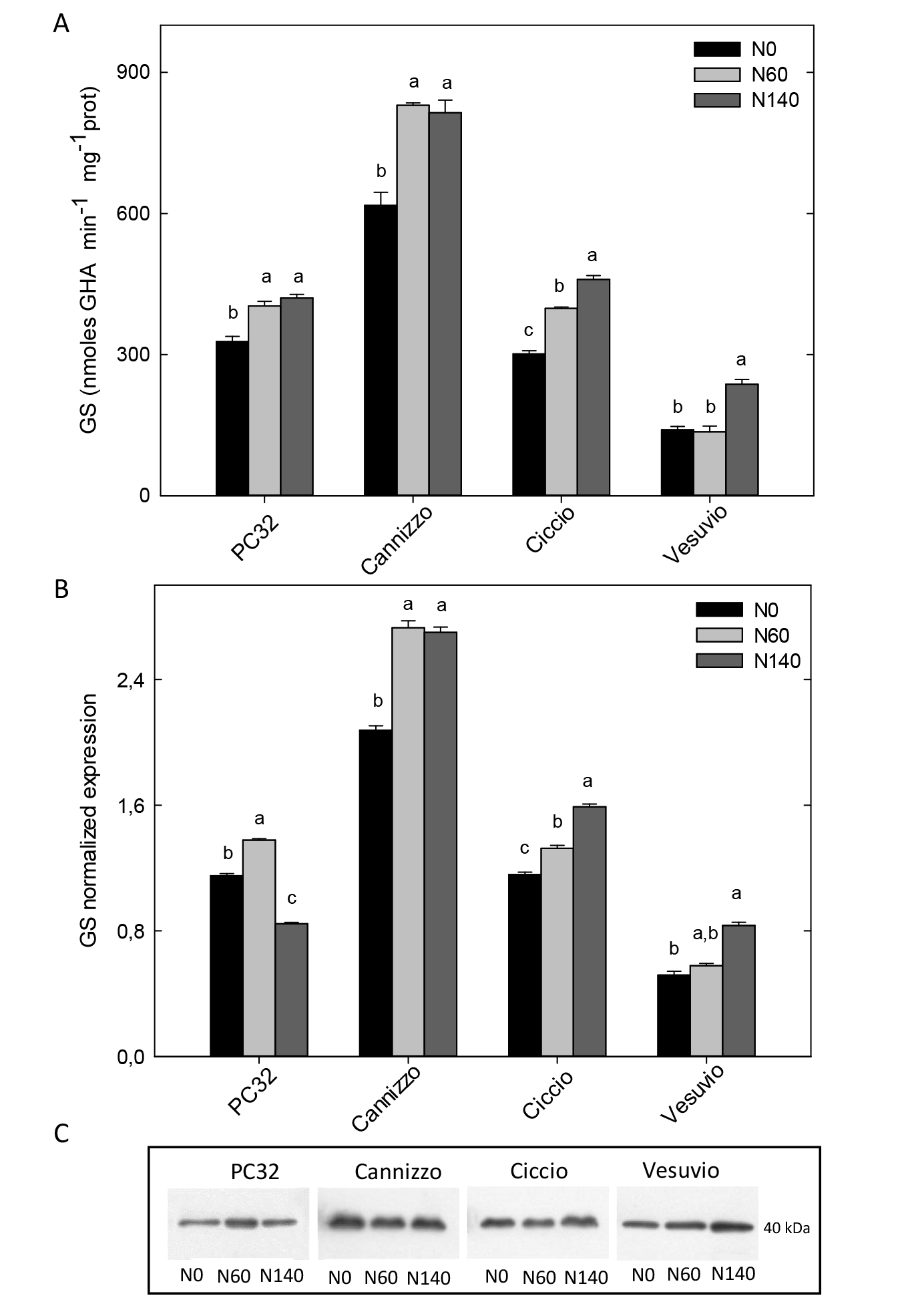

Supplement: Supplementary file 2 [file Image_2.TIF]

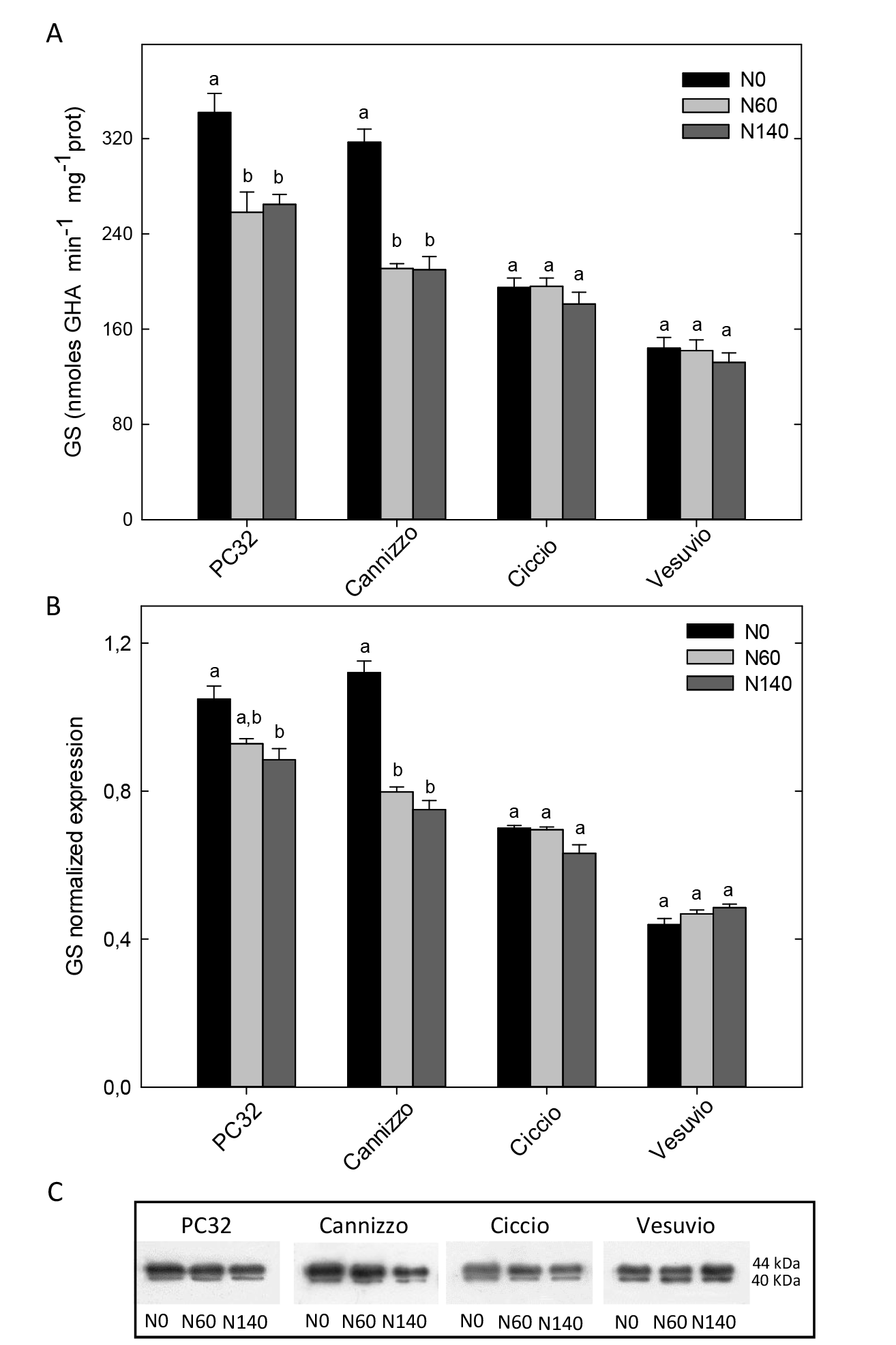

Supplement: Supplementary file 3 [file Image_3.TIF]

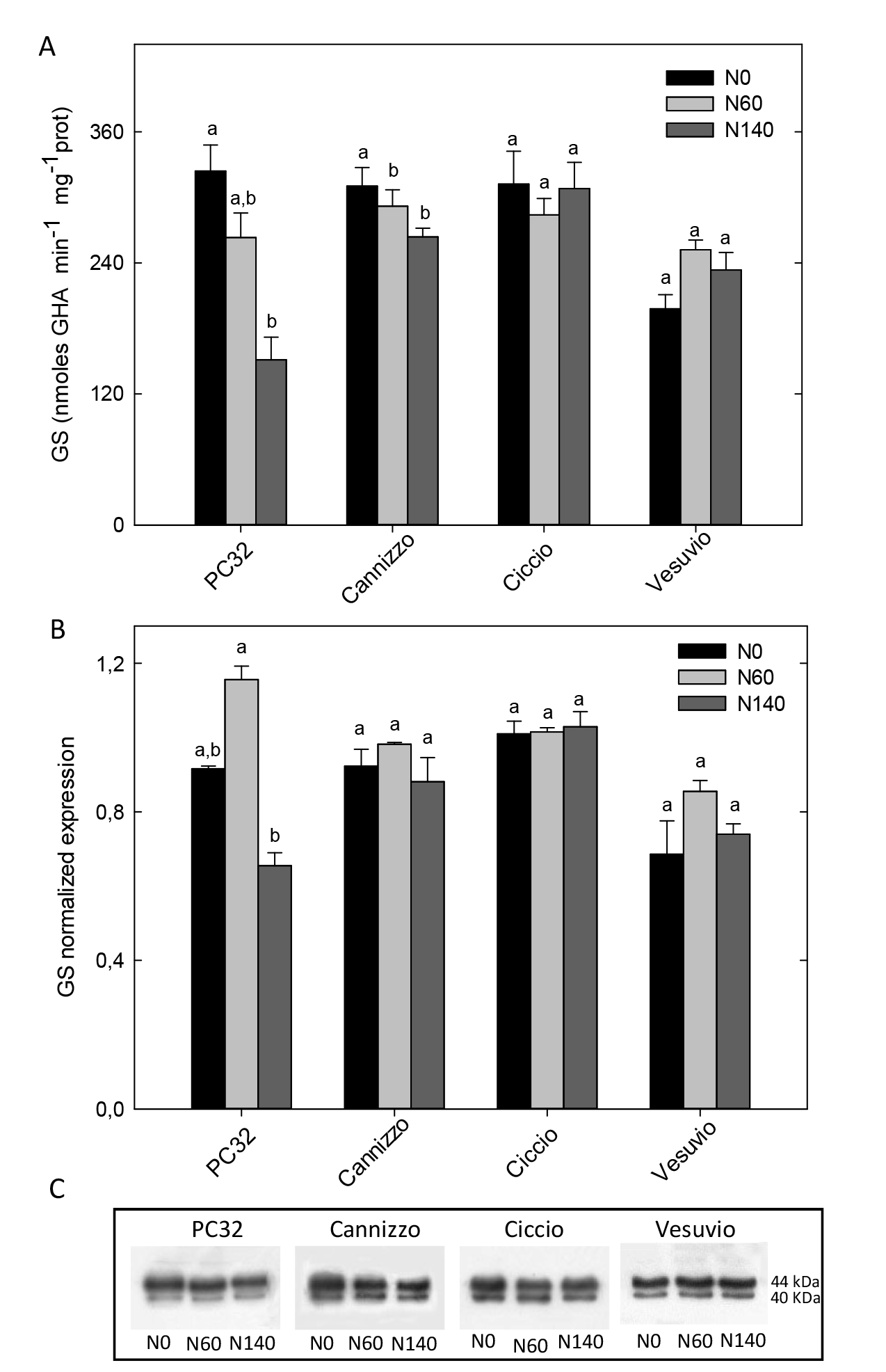

Supplement: Supplementary file 4 [file Image_4.TIF]

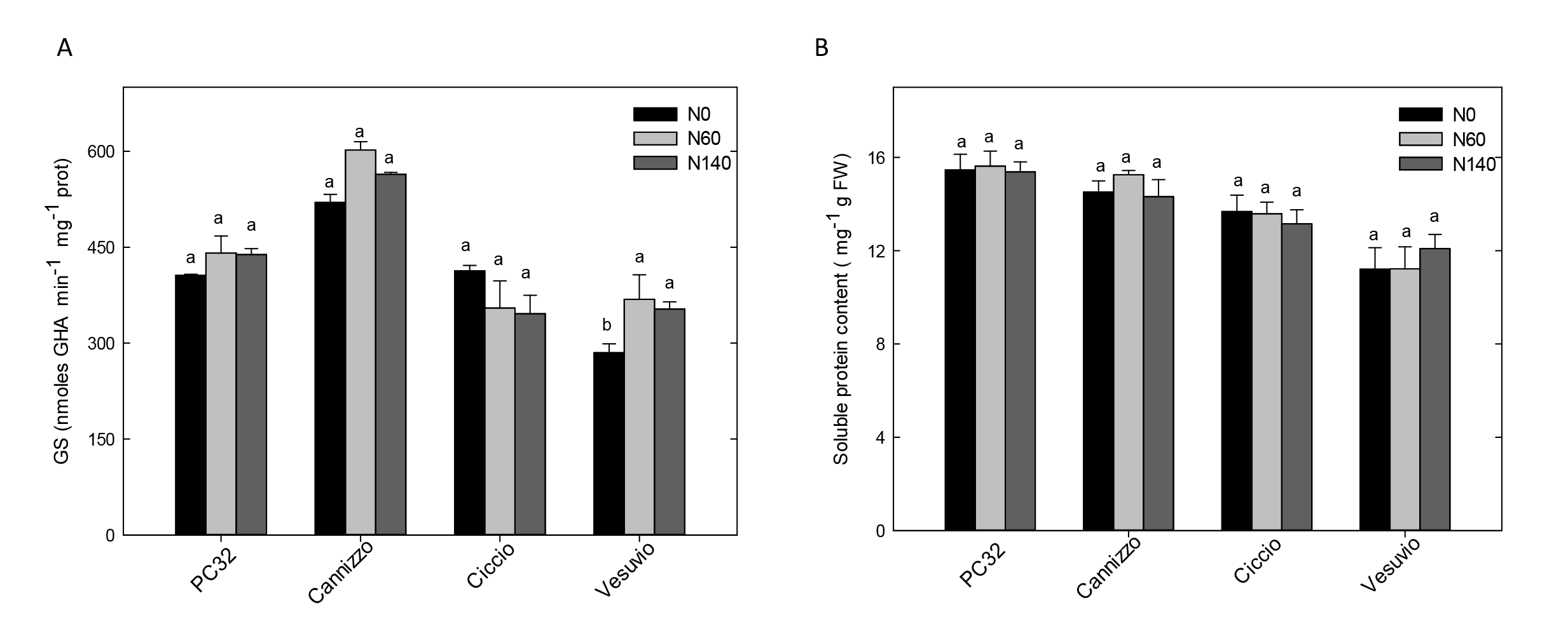

Supplement: Supplementary file 5 [file Image_5.TIF]
