# Supplementary material for: Glutamine synthetase in Durum Wheat: Genotypic Variation and Relationship with Grain Protein Content
Source: Front Plant Sci. 2016 Jul 13;7:971. doi: 10.3389/fpls.2016.00971 (PMC4942471; doi:10.3389/fpls.2016.00971)
Supplement: Supplementary file 6 [file Table_1.DOCX]

Suppl. Table 1 Grain protein Content (GPC), Grain Yield per Spike (GYS) and Thousand Kernel Weight (TKW) mean values of selected durum wheat genotypes grown for six years (2009-2013) at Valenzano (Bari, Italy). Values are the mean ± SE of the medium value obtained in each year; different letters indicate significant differences

(one-way ANOVA test; P ˂ 0.05);

|  |  |  |  |  |
| --- | --- | --- | --- | --- |
|  | **Genotype** | **GPC (g/100g DM)** | **GYS (g)** | **TKW** |
|  | Lucanica | 16.55^a^ | 2.09^a^ | 49.9^a^ |
|  | PI 191145 | 16.14^a^ | 2.03^a^ | 51.5 ^a^ |
|  | PC32 | 14.50^ab^ | 2.34^a^ | 50.8 ^a^ |
|  | Svevo | 14.37^b^ | 2.17^a^ | 52.1 ^a^ |
|  | Cannizzo | 14.51^b^ | 2.15^a^ | 51.2 ^a^ |
|  |  |  |  |  |
|  | Gianni | 12.74^c^ | 2.24^a^ | 49.8 ^a^ |
|  | Ciccio | 13.32^c^ | 2.21^a^ | 52.3 ^a^ |
|  | Appio | 13.12^c^ | 2.13^a^ | 51.7 ^a^ |
|  | Canyon | 12.51^c^ | 2.04^a^ | 50.9 ^a^ |
|  | Vesuvio | 13.18^c^ | 2.18^a^ | 51.3 ^a^ |
|  |  |  |  |  |
